# Aiguo Wu, Waheed S. Khan (Eds.): Nanobiosensors: from design to applications

**DOI:** 10.1007/s00216-020-02975-0

**Published:** 2020-10-09

**Authors:** Lothar Leidner

**Affiliations:** Eberhard Karls Universität Tubingen, Mathematisch- Naturwissenschaftliche Facultät, Institut für Physikalische und Theoretische Chemie, Arbeitskreis Prof. Gauglitz Auf der Morgenstelle 18, D-72076 Tübingen, Germany

**Bibliography**

Nanobiosensors: from design to applications

Aiguo Wu, Waheed S. Khan (Eds.)

Wiley-VCH

ISBN: 978-3-527-34510-6

Hardcover, 416 pages

March 2020, £125/€149.00

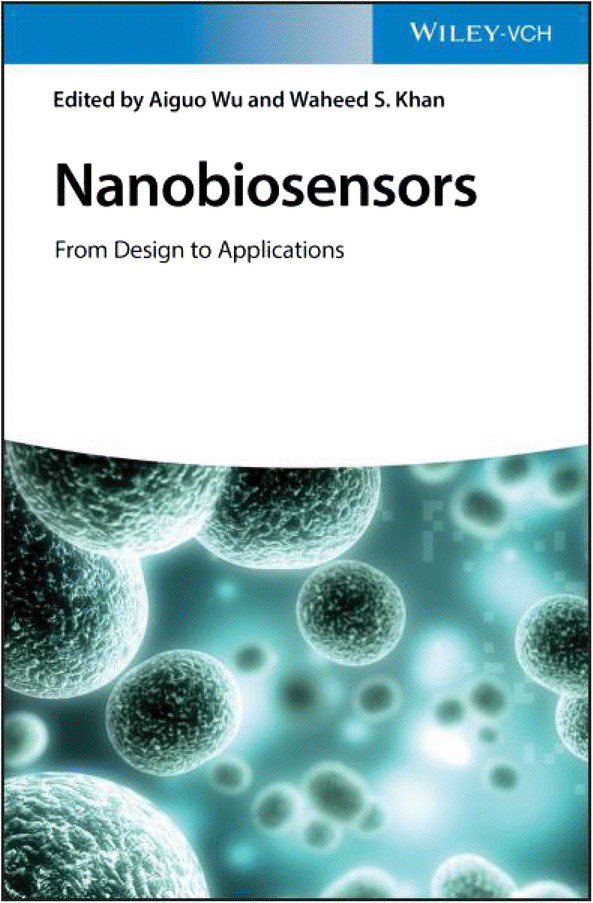



## Book’s topic

Nanobiosensors are used for the detection and sometimes quantitative measurement of bioanalytes. Applications range from biomarker detection and other diagnostics in medicine, epidemiology, environmental pollution detection, etc. The conventional way (and mostly the gold standard) to analysis is laboratory evaluation with well-known drawbacks like sample preparation (e.g., PCR), need of sophisticated equipment, and experienced personnel. Laboratory analysis is time-consuming and expensive. To circumvent these difficulties, different kinds of biosensors are being developed around the world. Biosensors produce fast results and are simple and inexpensive. Miniaturization reduces volume (and thus sample quantity). Physical laws in nanosized volumes often differ from their macroscopic counterpart.

*Nanobiosensors: from design to applications* gives an introduction into this world. Different kinds of analytic devices are presented, consisting of biological recognition elements and electrochemical, optical, piezoelectric, thermometric, or magnetic transducers. Important fields of application are introduced. Although not directly mentioned in the book, biosensors can contribute to COVID-19 epidemic research.

## Contents

The first four chapters focus on the basics of biosensor design and further development including new materials and biomarkers. The vast majority of the book deals with applications. Biosensors are designed to play a major role in clinical diagnostics, such as cancer and bacterial and viral detection including HIV. There are examples in the field of food quality (mycotoxins) and drug detection. Epidemiological issues are introduced in case of avian influenza and swine virus. Monitoring of environmental parameters, such as pesticides and marine toxins, is discussed.

Smartphone-based devices have great potential as point-of-care platforms for healthcare, food safety, environmental monitoring, and biosecurity. The final chapter reflects the use of smartphones in the field of bio-sensing.

## Comparison with the existing literature

Books about nanobiosensors exist since more than 15 years. To have a look into older ones gives some historical view of the progress made in the last decade and the changes in perspective. *Current trends in nanobiosensor technology*, edited by Bellan, Wu, and Langer (Wiley, 2011), falls into this category. In 2015, *Nanobiosensors and nanobioanalyses*, edited by Vestergaard, Kerman, Hsing, and Tamiya (Springer), was published, which gives a comprehensive overview of biosensors and their applications and future trends in the development. A newer book specialized in healthcare is *Advanced biosensors for health care applications advanced biosensors for health care applications*, edited by Inamuddin, Khan, Mohammad, and Asiri (Elsevier, 2019). Novel strategies for developing new systems to retrieve health information of patients in real time are presented.

## Critical assessment

There is a tremendous amount of research done in the field of nanobiosensors by a great number of international researchers. Looking at Google Scholar, one gets more than 10,000 references within the last decade. The fifteen chapters of the book at hand offer a wisely chosen, but necessarily subjective selection of sensor designs and applications introduced by experienced authors. More than thousand references are added for further reading. Nevertheless, it would have been enlightening, if the editors had mentioned criteria for their selection of topics and motivation in an introduction.

Because different chapters have been written by different authors, there is some redundant information in some places. This has the advantage, however, that the book does not have to be worked through from front to back, but can be read in the order of one’s own interest.

## Readership recommendation

The book gives an introduction for students, who want to get involved in the field of biosensors. As sensor development is a multi-disciplinary task, even the experienced researcher will find interesting information in fields slightly away from his own competence.

## Summary

*Nanobiosensors* gives an overview over many bio-sensing techniques based on nanotechnology. The book covers both sensing principles and their application in the fields of personal health care, drug discovery, epidemiology, and environmental issues. It offers an introduction for researchers, who want to get involved in the field of biosensors.

